# The power of public–private partnership in medical technology innovation: Lessons from the development of FDA-cleared medical devices for assessment of concussion

**DOI:** 10.1017/cts.2022.373

**Published:** 2022-03-10

**Authors:** Michael E. Singer, Dallas C. Hack, Daniel F. Hanley

**Affiliations:** 1 Vital Tech Partners, Bethesda, MD, USA; 2 University of Pittsburgh, Pittsburgh, PA, USA; 3 Virginia Commonwealth University, Richmond, VA, USA; 4 Acute Care Neurology, Johns Hopkins University, Baltimore, MD, USA

**Keywords:** Medical technology, concussion, medical devices, traumatic brain injury (TBI), medical technology innovation, medical device development, innovation, development

## Abstract

Given the convergence of the long and challenging development path for medical devices with the need for diagnostic capabilities for mild traumatic brain injury (mTBI/concussion), the effective role of public–private partnership (PPP) can be demonstrated to yield Food and Drug Administration (FDA) clearances and innovative product introductions. An overview of the mTBI problem and landscape was performed. A detailed situation analysis of an example of a PPP yielding an innovative product was further demonstrated. The example of PPP has led to multiple FDA clearances and product introductions in the TBI diagnostic product category where there was an urgent military and public need. Important lessons included defining the primary public and military health objective for new product introduction, the importance of the government–academia–industry PPP triad with a “collaboration towards solutions” Quality-by-Design (QbD) mindset to assure clinical validity with regulatory compliance, the development of device comparators and integration of measurements into a robust, evidence-based statistical and FDA pathway, and the utility of top-down, flexible, practical action while operating within governmental guidelines and patient safety.

## Introduction

Medical device development, from concept to US Food and Drug Administration (FDA) approval/clearance for product introduction into the market, can be challenging with a multitude of clinical, regulatory, technical, market, and financing hurdles to overcome [1]. Investing in the medical technology space can be uncertain when time frames vary and the bench to product to market pathway is usually tailored to the potential risks and benefits of any given device. Companies have often referred to facing the “Valley of Death” [2], the gap between idea and US market access; one solution is that some companies opt to focus on regulatory and product introduction outside of the USA, given a perceived less timely and expensive regulatory pathway [3].

Within the medical device sector, a unique product area that has established success in the transformation from idea to reality and access to the US marketplace has been technologies focused on traumatic brain injury (TBI) assessment. The Global War on Terror had created an urgent need to address its “signature injury,” mild TBI/concussion (mTBI) [4]. The innovation path for this product area had an unfortunately extensive history of failed trials in TBI and a reputation for clinical, technical, and regulatory difficulty both in neurology overall and TBI specifically [5,6]. Yet, beginning in 2009, private industry and the government, including both Department of Defense (DOD) and FDA, worked in a public–private partnership (PPP) to create medical devices focused on TBI resulting in several products eventually receiving FDA clearance. This included five different company/sponsor clearances through the FDA 510(k) *de novo* process, meaning that there was no predicate, a new FDA regulation would need to be established, and an FDA-approved, blinded multicenter prospective study would be required, as shown in Table 1.

**Table 1.** Food and Drug Administration (FDA)-cleared medical devices for assessing head injury through February 2022

Manufacturer	Device	Premarket database (FDA decision date)
Abbott Laboratories	I-STAT TBI Plasma Cartridge With The I-STAT Alinity System	K201778 (1/8/2021)
Anthrotronix, Inc.	DANA	K141865 (10/15/2014)
Banyan Biomarkers, Inc.	Brain Trauma Assessment Kit	DEN170045* (2/14/2018)
BrainScope Company, Inc.	BrainScope Ahead 100	DEN140025* (11/17/2014)
	Ahead 200	K143643 (5/15/2015)
	Ahead 300	K161068 (9/22/2016)
	BrainScope One	K181179 (5/18/2018)
	Modified BrainScope One	K181785 (12/19/2018)
	BrainScope TBI (Model: Ahead 400)	K183241 (2/19/2019)
	BrainScope TBI (Model: Ahead 500)	K190815 (9/11/2019)
ImPACT Applications, Inc	ImPACT	DEN150037* (8/22/2016)
		K170209 (2/23/2017)
	ImPACT Quick Test	K170551 (6/21/2017)
	ImPACT	K181223 (10/20/2018)
	ImPACT Version 4	K202485 (12/25/2020)
InfraScan, Inc.	Infrascanner Model 1000	DEN100002* (12/13/2011)
	Infrascanner	K120949 (1/11/2013)
	Infrascanner	K200203 (7/10/2020)
	Infrascanner	K211617 (2/9/2022)
Oculogica, Inc.	EyeBOX	DEN170091* (12/28/2018)
	EyeBOX	K201841 (9/6/2020)
	EyeBOX (Model EBX-4)	K212310 (12/22/2021)
SyncThink, Inc.	EYE-SYNC	K202927 (10/2/2021)
Vista LifeSciences, Inc.	ANAM Test System: Military Battery	K150154 (8/28/2015)
	ANAM Test System	K201376 (3/25/2021)

* “DEN” denotes FDA 510(k) *de novo* classification.

To highlight the opportunities for medical device development, mTBI presents both challenges and solutions that have led to several products authorized for the US marketplace. This review includes an FDA-cleared medical device for the assessment of both structural and functional brain injuries associated with mTBI. Such a partnership involved a US-based private company (BrainScope Company, Inc.) working with R&D funding from the US DOD to bring regulatory submissions to the FDA. Over the course of nearly a decade, DOD funded and supported multiple research contracts which resulted in several subsequent FDA clearances; in these contracts, the company followed a robust, evidence-based regulatory pathway to FDA clearance and eventual introduction by the company of a novel, handheld, ruggedized, multimodal, medical device incorporating AI-derived algorithms to aid the clinician in objective, rapid assessment, and diagnosis of mTBI. These AI-derived algorithms, of course, did not come without risk; clinical validation was necessary in order to prove accuracy [7]. The PPP between DOD and the company further illustrated a financial symbiosis and mutual leveraging which benefitted the healthcare mission of each party.

The challenges and solutions from this example of medical device innovation of mTBI assessment technology through PPP from development to regulatory clearance to market introduction are instructive for important untapped medical conditions outside the more traditional areas of the body, particularly those with little to no prior objective capabilities.

## An Important Intersection between mTBI and Medical Devices: US Service People and Organized Athletics

Often referred to as a “silent epidemic,” 69 million individuals worldwide are estimated to sustain a TBI each year [8]. Roughly 2.8 million people in the USA visit hospital emergency departments (EDs) each year due to TBI [9,10]. The topic of mTBI in organized athletics has been a national conversation in the USA. Even further, on an international scale, TBI became the “signature injury” of the “Global War on Terrorism” [11] following the attacks on September 11, 2001 [12]. Different than in previous conflicts and wars, the advent of body armor protected warfighters from substantial visible body harm, which in the past led to mortality. This created a new problem: while in prior conflicts the patient would have likely died, instead the exposed human brain is uniquely damaged. Further, enemy improvised explosive devices (IEDs) were a weapon of choice beginning as early as 2003, causing “blast injury” to the brain not extensively experienced in prior wars and with seemingly a different pathophysiology than blunt-force head trauma [13]. Brain-injured warfighters experienced a range of TBI from mild to severe, with mTBI being the predominant level of injury, estimated to represent 86% of these injuries. Identification and assessment of TBI, particularly its milder forms, remained subjective and based on physician qualitative assessment and without an objective assessment aid tool [14].

In the civilian environment, sports-related forms of mTBI (concussion) were part of the "national conversation" emanating from the popularity of football, soccer, hockey, and other sports, particularly at the high school level [15]. Media coverage increased awareness of potential long-term hazards of head injury and successive head impacts, including dementia, chronic traumatic encephalopathy (CTE) and amyotrophic lateral sclerosis (ALS) [16,17]. Indeed, public pressure brought about by the visibility of those diagnosed with CTE, including professional football players, likely contributed to the motivation behind the PPP in TBI.

Computerized tomography (CT) scan remained the traditional assessment course for head-injured patients in urgent and emergency situations as a rule out for life-threatening brain hemorrhage, including various forms of hematomas. While the literature indicated 82% of head injured patients were referred for a head CT scan in the ED, 91% of these scans were found to be normal or negative for severe injury [18]. This practice led to substantial overuse of CT scanning and is why the American College of Emergency Physicians (ACEP) named “Head CT” as the most overused, and often unnecessary, medical technology [19].

The direct and indirect costs of the broad spectrum of TBI among Americans, regardless of their military status, were reported as $76.5 billion annually [20]. Other socially driven economic impacts such as substance abuse and homelessness were not considered, likely greatly underestimating the overall economic burden of TBI. The broad under-recognized scope of the brain injury problem was further underscored by the human psychology often associated with team activities seen in warfare theater and sports fields with brain-injured patients being unwilling to admit to their injuries for fear of letting down their teammates.

Without objective assessment and diagnostic capabilities, assessment of such injuries depended on subjective self-reported signs and symptoms. Further, such evaluations were often performed by physician specialties unrelated to the brain, such as sports medicine and orthopedics, and with the pressure from operational leaders (such as military commanders or coaches) overhanging their clinical decision-making’s impact on military-related missions or competitive games despite the potential long-term consequences to the head-injured patient.

## The Mild TBI Innovation Challenge – Characterizing mTBI and Establishing Safe and Effective Medical Technology

With such a substantial market need involving public awareness and pressure, addressing both diagnostics and therapeutic issues became an obvious area for entrepreneurialism as well as a military and academic partnership focus.

The difficulty of successful medical technology advancements in the neurology space, well outside of the more traditional medical device success areas in cardiology and orthopedics, compounded already complex technology and clinical challenges and made success rare. TBI was also well established in academic circles as a haven for failed trials. The tangible failure in the area was often thought to be a result of poor clinical trial design, creating substantial academic skepticism [21].

Yet substantial recognition that medical technology directed to the brain as “the last frontier” and “grand challenges of the 21st century” became increasingly understood and visible, with the White House launching the BRAIN (Brain Research through Advancing Innovative Neurotechnologies) Initiative in 2013 [22]. Notably, the BRAIN Initiative dedicated a focus specifically around TBI/head injury with a consortium of stakeholders focusing on advancing scientific and clinical progress for service men and women [23].

## Initial Focus on Diagnosis from the Military Need for Rapid Diagnosis in the Field, with TBI as the “Signature Injury” of the “Global War on Terrorism”

Beginning in the 2008–2009 timeframe, roughly six years after the 9/11 attacks and the advent of the Global War on Terror, the US DOD began a substantial review of potential diagnostics and therapeutics related to the full spectrum of TBI with a competitive presentation of roughly a dozen promising technologies to experts held at the annual meeting of the 13th annual Advanced Technology Applications for Combat Casualty Care (ATACCC) conference. The US Army Medical Research and Materiel Command (MRMC) publicly commented about their views on collaboration when they stated, “Military medicine is often at the forefront of medical research. Often, the needs of Soldiers are identified and through collaborative efforts, those needs directly evolve into products licensed by the US FDA. Many can later be found in civilian emergency rooms protecting public health” [24]. Moreover, while remaining the regulatory oversight body for medical devices, during this time FDA espoused its Critical Path Initiative through “the use of public-private partnerships and consortia” [25], including to address the development gap to “create cross-industry and cross-agency (FDA/NIH/CMS) collaborations to evaluate multiple technologies” [25] generally in drug, device, and diagnostics development.

With substantial public awareness, DOD focused its initial efforts on creating an objective diagnostic framework, sorely needed both for clinical and research purposes for the development of therapeutics. The range of candidate diagnostic technologies included near-infrared, eye tracking, blood-based biomarkers, neurocognitive tests, and electroencephalogram (EEG) (see Table 1).

In 2009/2010, BrainScope Company, Inc. presented early data to DOD’s Combat Casualty Care Research Program and US Army Office of the Surgeon General showing potential accuracy for identification of mTBI. Although the company had received a traditional 510(k) for a handheld EEG reader capability with real-time artifact recognition in 2009 [26], there was no specific indication for applicability to mTBI. As a result, the company (recognized as “the Sponsor” to FDA) decided to pursue an algorithm development study to create a brain activity biomarker, followed by an independent validation study to demonstrate statistically significant efficacy to identify the likelihood of a structural brain injury (brain bleed).

As discussed in the following section, decisions were made relating to its regulatory path – eventually the 510(k) process [27] – which critics of the current regulation have argued adds delays to introducing new devices to market without improving patient safety [28], while proponents support the process to ensure safety and efficacy [27].

**Fig. 1.**
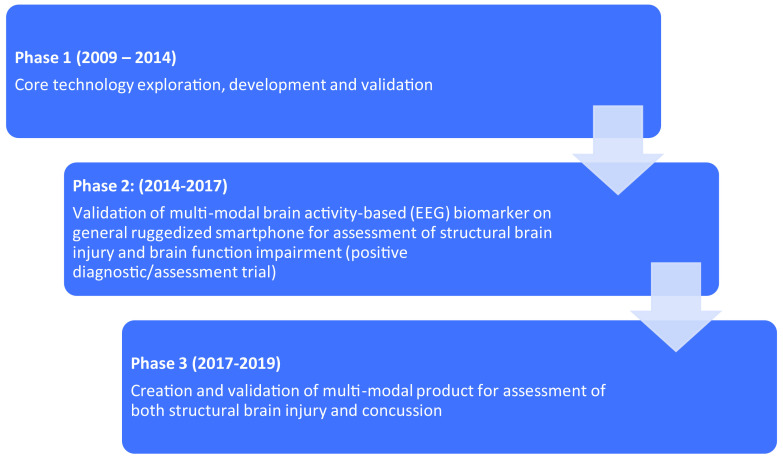
Public-private partnership (PPP) Pathway to Create mild traumatic brain injury (mTBI) Diagnostically-Focused Product.

In a case study, there were three distinct phases of the PPP among industry, government, and academia to create a diagnostically focused product for mTBI (Fig. 1).

Each phase is discussed below.

### Phase 1 (2009–2014): Core Technology Development and Validation – The Exploratory Phase of Medical Device Development

While the initial technology had been the product of several years of translational research pursuits, there remained many TBI-related technology challenges. The specific clinical trial correlates profile for what might be considered an mTBI, or in statistical parlance “truth” or clinical gold standard, needed to be clarified to execute a clinical trial. The regulatory pathway had not been clarified. And questions remained as to how a long-questioned modality, EEG, could be easily and rapidly applied to the individual in any environment, including “noisy” venues at the point of care, outside the established EEG use area of epilepsy [29]. An obvious question was: could EEG be demonstrated to be accurate and have sufficient efficacy to be useful for a different, more common medical condition which can be life-threatening and often is life-altering?

A key decision in the development of the product included a decision relating to the correct FDA pathway. Despite mTBI being the most common among various forms of TBI, there was neither a clear-cut clinical gold standard nor a precedent with specific FDA labeling relating to mTBI. Two companies had received FDA 510(k) clearances before/during this period (2009–2014) – a *de novo* 510(k) (with no previous predicate) relating to hematoma detection (“Infrascanner”) [30] and a traditional 510(k) (with a predicate) for “objective measurements of reaction time (speed and accuracy) to aid in the assessment of an individual’s medical or psychological state” (“DANA”) [31]. Neither, however, had FDA Indications for Use (IFU) specific to “brain injury,” “TBI,” “mild TBI,” or “mTBI.” BrainScope had early evidence that such a capability could be demonstrated.

In determining whether to proceed with a 510(k) or Premarket Approval (PMA), it was clear that neither path presented a natural option. On the 510(k) side, there was no “predicate.” And on the PMA side, the lack of an obvious, consensus gold standard encapsulating the entirety of the pathophysiology, including the clinical correlates to (considered a functional injury), was a major limitation. It was clear that the appropriate device classification was Class II, and hence the regulatory pathway would need to be *de novo* 510(k). While the 510(k) pathway was the more expedited of the two medical device regulatory pathways in comparison to PMA, a blinded multicenter prospective clinical study would be required – irrespective of military support for the project – to provide sufficient evidence to create a *new* predicate (as the term *de novo* indicates), creating a new class of 510(k) regulated products. Moreover, given the novelty of unmet need, it was determined and publicly announced that this situation warranted what was then called “Expedited Review,” now called “Breakthrough Designation” [32,33].

To design such a practical study, it was necessary to establish definitions for both “clinical truth” and device-related diagnostic truth emanating from an agreed standard of care. Determining this clinical truth in mTBI patients was a major focus of this effort, with the Glasgow Coma Scale (GCS) and the CT scan as the two center points. GCS, the traditional rapid assessment of severity of TBI [34], was used as a proxy to determine inclusion of patients presenting with mTBI at a level of 13–15 (out of a possible 15). Thereafter, the standard of care for patients who are head-injured with a higher GCS (e.g., 13–15) are typically referred for a CT used to visualize and rule out “structural injury” such as skull fracture, tissue edema, and/or presence of intracranial bleeding, which, if undetected, can have life-threatening consequences. CT machines are primarily located in hospitals adjacent to or within EDs and are not practically useful in venues requiring timely results outside of institutional medical facilities (such as outside in military theater or sports venues). While CT scans were the standard of care, there was evidence in the peer-reviewed literature that CT scan reads had medium to poor inter-rater reliability [35]. Nonetheless, it was determined to use CT as the standard of care to determine “clinical truth.” Further, to improve reliability of the reads of CT images, consensus of an independent, blinded panel of three expert neuroradiologists would be required.

A CT scan provided important advantages for the clinical study, such as the ubiquity of the technology and the ability to conduct a trial without imperiling clinical flow. However, head injured patients with a GCS score of 15 were not always CT scanned, leaving the question as to whether a patient in whom clinical judgment was not to obtain a CT could be assumed to be CT-negative. Feedback from clinical investigators, Institutional Review Boards (IRBs) at the participating institutions regarding exposing a patient to CT radiation when not clinically indicated (including clinical judgment), which might include use of a clinical decision rule such as the New Orleans Criteria (NOC) or Canadian CT Head Rule (CCHR) and incorporating a 72- to 96-hour follow-up to assure determination, supported deeming such patients CT negative. This assumption reached eventual consensus among all parties [33]. As such, this important group of the most mild head injured patients were included in the study. It was also agreed that the product could not replace a CT scan but would provide objective information which could lead to a CT scan, hence creating “an adjunctive” tool for mTBI. The term “assessment,” moreover, was used in lieu of “diagnosis.”

The largest problem with CT as “clinical truth” is that CTs can only show “structural injury,” thereby not only limiting the potential FDA IFU for the product, but also imperiling potential performance in the clinical study of patients with TBI induced aberrant profile of EEG yet normal CT scans, creating a situation where the gold standard – CT – could be less sensitive than the instrument being assessed [36]. In this scenario, the disruptions in brain function would not be appropriately identified. Magnetic resonance imaging (MRI) was considered as a potentially more precise alternative to CT as the clinical study gold standard given its enhanced capability to capture some functional TBI pathologies, but given MRI is not practically available in the ED and takes longer to perform, it was deemed both impractical and more importantly, inconsistent with current clinical practice.

The efficacy of the device truth, relative to the CT “clinical truth,” was established using a receiver operator curve (ROC), demonstrating the performance of the classification model (device truth) at all classification thresholds plotting two parameters: the True Positive Rate (Sensitivity) versus False Positive Rate (Specificity). Perfect performance would be 100% Sensitivity and 100% Specificity. Of course, this level of perfection is unrealistic in human diagnostic tests, so trade-offs between Sensitivity and Specificity (“stratification of risk” [37]) are paramount in the selection of the classification threshold. Given the risk that a False Negative (undetected potential bleed) could potentially be life-threatening, or lead to a patient not receiving a needed CT scan, the focus in performance along the ROC curve was on Sensitivity or Negative Predictive Value (NPV) rather than Specificity or Positive Predictive Value (PPV) [38]. Such is not always the case in diagnostic medical technology [39] but is often the case in life-threatening diagnostics. This stratification of risk was also consistent with the fact that current clinical practice was to scan ∼85% of head injured patients, and as such Specificity was not the main concern of the clinicians. However, Specificity above that of current CT clinical decision rules (e.g., NOC or CCHR) was desired, as it would increase precision and potentially help reduce unnecessary head CT scanning.

In the validation study, a total of 817 subjects were enrolled at 11 study sites in the USA and were included in the intent-to-treat (ITT) population [40]. The trial proceeded in two phases: algorithm development and validation. The initial algorithm development phase of the study was completed, and using machine learning/AI methodology, a final proprietary algorithm was derived and then validated in the second phase, identifying a specific set of features characterizing the EEG that optimally separated the CT+ and CT− populations [41], establishing a brain activity-based biomarker for identification of the likelihood of a CT+ finding.

For purposes of validation, measurement of the presence of hematomas, including location and size of the blood pool identified from the CT DICOM images as established by the independent blinded Johns Hopkins BIOS image laboratory, demonstrated strong accuracy in those patients with even the smallest detectable level of blood regardless of the location or size of the blood pool; and NPV in the clinical investigation was very high, demonstrating the very high likelihood that patients with a negative device classification had no structural injury identifiable on head CT. The market need and sufficiently strong statistical evidence with a focus on NPV/Sensitivity, especially to the presence of measurable blood, was sufficient for a 510(k) *de novo* classification for a new product called “Brain Injury Adjunctive Interpretive Electroencephalograph Assessment Aid,” which was established on August 19, 2014 [40].

### Phase 2 (2014–2017): Validation of Multimodal Brain Activity-Based Biomarker on General Ruggedized Smartphone for Assessment of Structural Brain Injury and Brain Function Impairment

Despite an FDA clearance [40], both the performance of the device in the validation study and the form factor (e.g., design usability) of the device itself led to a decision not to commercialize this version of the device. On September 30, 2014, the company announced further substantial DOD funding for the next generation of products, recognizing the promise of the technology [42]. Learnings from prior clinical studies, including the validation study, and advances in the field of mTBI indicated a need for a proprietary “multimodal” [43] marker which, while using EEG at the core, included selected clinical features. Leveraging substantial advances in signal processing, real-time processing capabilities, artifact detection [44], and use of machine learning/AI algorithm methodologies facilitated the creation of proprietary next-generation replicable algorithms to identify EEG-based biomarkers of TBI [45]. In addition, the ubiquity of smartphones used as medical devices became more omnipresent with the challenge of having a single-use smartphone sufficiently ruggedized for challenging environments.

As the company embarked on further clinical studies to improve performance necessary for a marketable product, with the benefit of the initial *de novo* predicate in place [40], the company used a regulatory 510(k) laddering strategy in which each 510(k) clearance created a step to the next upgraded submission and clearance. This route of building on a 510(k) was (and remains) typical, and no other regulatory route (e.g., PMA) would be needed. Using this regulatory laddering, the first subsequent 510(k) was for the earlier *beta* hardware capability to be applicable to a generalized and ruggedized smartphone [46]. Further, recognizing the need for not only a multimodal algorithm, but a battery of capabilities for full assessment of the head-injured patient, other capabilities such as rapid neurocognitive testing and digitized standard concussion assessment tools were added in a subsequent 510(k). Importantly, the use of EEG could also be used to indicate overall “brain function,” a broad indicator of functional brain impairment.

In the second validation study [40], the target population and protocol were similar in all aspects to the prior *de novo* 510(k) study, with the primary end points, as defined by Sensitivity and Specificity of the study device classification distinguishing CT+ from CT−, successfully exceeding the performance goals with demonstrated significantly improved performance, with Sensitivity to ≥1 mL of blood, and a high NPV. This study was an independent validation of an *a priori* EEG-based biomarker of an mTBI algorithm and included three independent CT scan reviewers [40]. Repeatability and reproducibility of the device result were also successfully demonstrated [40]. There were three categories of output with respect to the likelihood of CT results: Positive, Negative, and Equivocal. The equivocal category incorporated a small percentage of patients near to the positive classification threshold to be called out, just as with medical practice of identifying “pre-diabetes” or “pre-hypertension,” importantly moving beyond the binary classification of the prior device. In addition, using the same EEG recording, a new “Brain Function Index” was added to “reflect the evolution” of the product to include an indicator of brain function impairment, and an FDA-cleared capability as an element of the battery of tests [47].

An FDA clearance was granted on September 22, 2016 [48], and a first of kind product for assessment of mTBI launched in early 2017, which led to the product’s nominations for the Prix-Galien Award in 2 successive years [49]. An investigator-initiated peer-reviewed publication appeared in *Academic Emergency Medicine*, detailing the derivation and validation of the device [40].

### Phase 3 (2017–2019): Creation and Validation of Multimodal Product for Assessment of Both Structural Brain Injury and Concussion

While the market introduction of an mTBI product focusing on structural injury and brain function impairment at the point of care as an adjunct to triage of head injury was well underway, DOD had also funded BrainScope to create a product for assessment of “concussion,” a Concussion Index (CI) including longitudinal assessment, going beyond the acute assessment focus of the existing algorithms. Yet there were different challenges which were successfully addressed for this product spanning clinical, product feature, and regulatory elements.

The clinical study challenges were addressed through a multisite clinical study, which included assessments preseason in one cohort; an injury cohort with assessments at time of injury and follow-up assessments at an intermediate point in recovery at clinically determined return to play (RTP) and at 45 days after RTP. The multiyear longitudinal study was funded from research contracts from the DOD and the National Football League (NFL), GE Head Health Challenges (I and II), and private investment. Despite funding from the DOD and NFL, there was no direct or indirect sway on the clinical study design, protocol, or results.

The product feature challenges primarily included the creation of an algorithm using multimodal features which could prove sufficiently stable and replicable in controls over time and reliably reflect change over time in the concussed population. This algorithm derivation required a substantial dataset of patients and controls for use in the machine learning/AI methodology for the identification of an EEG-based biomarker of concussion [50].

The regulatory challenge was the lack of a traditional gold standard for assessment, such as CT was for “structural injury” in prior FDA-cleared products. The gold standard for diagnosis of concussion would be based on the clinical site guidelines – all fundamentally NCAA guidelines – to offer the greatest ability to generalize results. Throughout 2018–2019, progressive additions of descriptive language related to the product in successive, laddered 510(k) clearances. In particular, the term “concussion,” once considered a layperson’s term, was added to the IFU in early 2018 [51], followed by adding the terms “multimodal, multiparameter” in late 2018 [52].

The CI validation study, an external validation test of an *a priori* CI algorithm, was performed on 580 subjects across 10 US clinical sites including high schools, colleges, and concussion clinics [53]. The validation study achieved and exceeded the prespecified performance targets for the primary end points. Analysis of the study results further demonstrated that, at a population level, stability of the CI in the non-head injured population studied over time, allowing reliable interpretation of change in CI over time in the injured/concussed population (reflecting recovery). An investigator-initiated, peer-reviewed publication appeared in *JAMA Network: Online,* detailing the derivation and validation of the CI [53].

On September 11, 2019, the “Concussion Index” was cleared by the FDA [53,54], almost 10 years since the ATACC meeting in 2010, adding to the multimodal capabilities including the Structural Injury Classifier and the Brain Function Index. Over the course of the years, a substantial Intellectual Property portfolio with over 100 issued and pending patents on the technology had been developed. Poignantly, 18 years – nearly two decades – had passed since the attacks of 9/11, yet DOD in partnership with industry, academia, and its governmental regulatory counterpart the FDA, had stuck with the PPP mission of creating a diagnostic toolbox for TBI, the signature injury of the global wars on terror, through funding and clinical, technology, and regulatory innovation.

## Key Lessons in Medical Technology Innovation

Ten key “Critical to Quality” lessons are highlighted below from this review, driven heavily from a quality lens, including examples gleaned from the Quality-by-Design (QbD) literature and general facets of “continuous improvement” and “culture of quality” found in the literature on Learning Health Systems (LHS) responding to the needs of the at-risk population [55].

1. **The primary “public (and military) health” objective as a driving force for medical device development.** The advancement of TBI diagnostically focused technologies was based on the core principle of addressing public and military health. Given the DOD’s need for a diagnostic capability amidst the Global War on Terrorism which could address its TBI “signature injury” problem, the military helped to substantially advance a substantial civilian medical issue as well. With this lens, the utilization of a generalizable, objective clinical evidence [56] from all parties led to successful outcomes.
2. **Government and academia “collaboration towards solutions” with industry.** The government, specifically the DOD, brought financial resources for product requirements, while the FDA worked constructively on appropriate regulatory pathways. Academia, along with an academically based Contract Research Organization (CRO) implemented clinical studies and a diagnostic trial design structure in a methodical fashion, specifically algorithm analysis, precision measurement of a “gold standard comparator,” clinical pragmatism, trial design inclusion criteria, and core lab standardization. The choice of an academically oriented CRO was based on three core concepts: the combined clinical and technical capabilities specific to the clinical trial at hand, specifically the technical capabilities to methodically interpret brain hemorrhage, a scientific and clinical lens unique to academic CROs, and a validity that academic institutions bring to regulatory discussions with FDA. Industry executed with a business orientation towards creating a product for market. Such a focus on team partnership with a QbD lens of “collaboration towards solutions” [57] (incorporating the government–industry–academia PPP triad) is more broadly demonstrated through the Clinical Trials Transformation Initiative (CTTI) shown in Fig. 2.
  **Fig. 2.**
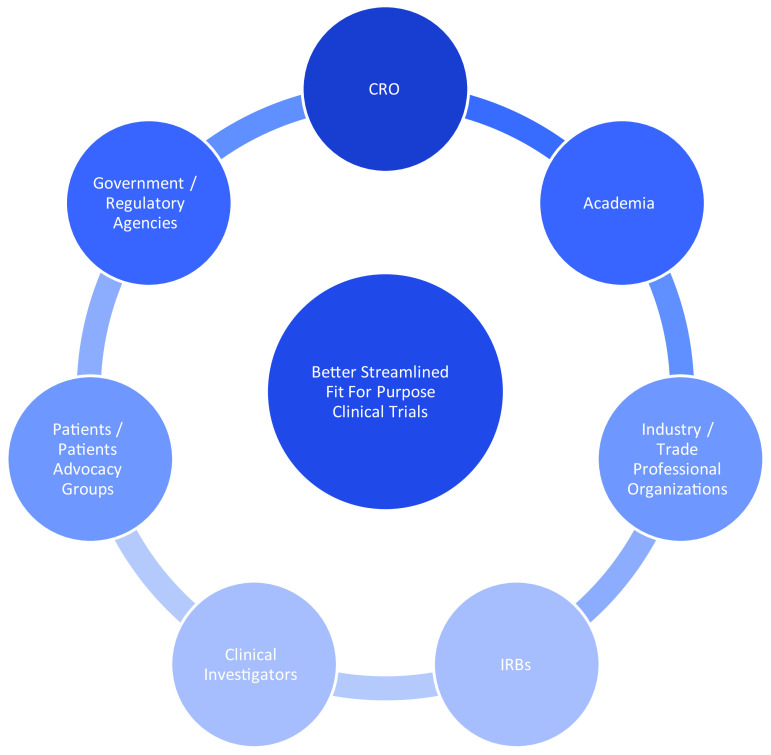
Clinical Trials Transformation Initiative (CTTI): “Collaboration Towards Solutions.” Source: Clinical Trials Transformation Initiative, *Clinical Quality-by-Design (QbD): Principles to Practice*, August 21, 2015, Page 7. CRO, Contract Research Organization; IRB, Institutional Review Board.
3. **DOD Industry Symbiosis and Mutual Leveraging.** The substantial partnership between DOD and the company further illustrated a financial symbiosis and mutual leveraging which benefitted each party. As DOD contributed research funding, the company matched and surpassed this funding with its own private funding, thereby aiding in the mission for both parties. Auspiciously, in a December 2021 speech, Secretary of Defense Lloyd Austin noted "the valley of death" small companies face to progress from “idea from inception to prototype to adoption by the department,” and as such DOD would be “‘doubling down’ on efforts to help small businesses and other innovators bring new technology to the military” [58].
4. **Embracing an evidence-based “Culture of Quality” in clinical trial management, regulatory relations, and biostatistics.** As CTTI describes, “‘quality’ is defined as the absence of errors that matter to decision-making – that is, errors which have a meaningful impact on the safety of trial participants or credibility of the results (and thereby the care of future patients)” [57]. Embedding a three C’s focus on Continuous Improvement, Competence, and the Customer, such quality measures, with benefit from the LHS literature, led to tangible medical device-specific quality certifications, such as ISO 13485 [59] and industry best practices recognition [60].
5. **Consistent regulatory focus and the benefit of progression of advancement through interactive review with FDA.** The company as “sponsor” worked with the FDA in an interactive manner, leading to identifying opportunities to expedite from ideas to patient translation through pathways, including pre-IDE concurrence, interactive review, and progressive FDA clearances supporting the natural evolution of a product. As an example, a joint working group, including FDA officials, wrote with respect to care for intracerebral hemorrhage:
  1. Regulators are responsive to medical needs and public health imperatives, and regulatory pathways provide effective means to expedite products to patients. More can be done to use and further develop expedited and adaptive regulatory pathways within the existing regulatory framework to allow patients to have early access to new treatments. The development and refinement of such pathways requires ongoing dialog and collaboration between the regulatory bodies, such as the FDA, Center for Medicare and Medical Services, patient groups, and leaders from industry and academia [61].
6. **Development of device comparators and integration of measurements into statistical (analytic) and FDA pathway.** The success of this medical device pathway illustrated the benefit of identifying early in the interactive process with FDA a specific gold standard – CT in this case – and using this measurement for assessment of injury characteristics. The development path evolved such that the measurement of injury characteristics was successfully implemented in clinical trials, creating vital evidence leading to multiple FDA clearances.
7. **Using physician and patient input as the Voice of the Customer (“VOC”) [62] for product development.** The product introduced to the market illustrates the benefit of attaining vital input from physicians and patients, which focused in four areas:
  - incorporating a proprietary multimodal capability [43] and approach included in FDA labeling [52] for complex pathological situations such as mTBI/concussion, where various clinical inputs physicians are accustomed to use become vital “features” to offer a multiparameter capability;
  - the size and durability of the handheld device in different, sometimes adverse environments;
  - the on-screen output language incorporated in the various FDA clearances; and
  - the application of the headset on patient foreheads in which application would be rapid and reliable and not painful or intimidating to the patient [63].
8. **Terminology specification, accuracy, and harmonization to characterize a device’s capabilities crucial for FDA approval/clearance.** With broadly accepted clinical standards and definitions for mTBI laden with field disagreement, a consensus-building process for terminology harmonization among the various constituencies was vital for description and definition of key terms (and their relevant clinical parameters) for the protocol and clinical trial end point finalization. The main effort at terminology harmonization for TBI was the Common Data Elements initiative led by the NIH and DOD [64], which matured after the BrainScope process. In this circumstance, the “Structural Injury Classifier” (for structural brain injury) [40], the “Brain Function Index” (an index measuring acute brain function impairment) [47], and the “Concussion Index” (an index specific to “concussion”/mTBI and not limited to the acute application) [50,53] illustrate the benefit of appropriate and accurate terminology delineating a device’s capabilities.
9. **Creating ongoing scientific and clinical evidence and acceptance through publication via the development of a consortium of university leaders from the outset.** The company aggregated leading clinical thought leaders in emergency medicine, neurology, university-supported researchers, and sports medicine and concussion, vital to create evidence-based scientific proof and a library of peer-reviewed literature important for overall clinical acceptance. FDA and clinical thought leaders are wary of any “black box” technology, so it became vital to publish technical capabilities, particularly those related to multivariate algorithms, in the peer-reviewed literature [45].
10. **Shared purpose for the utility of top-down, flexible, practical action while operating within governmental guidelines and patient safety (minimal risk)**. The PPP among DOD, FDA, academia, and the company was practically minded, realizing that there remained a substantial clinical need. The company “sponsor” followed the requirements for designation and clearance of *de novo* 510(k) products, and at no time experienced preferential treatment by the FDA. Clearly delineated evidence-based clinical information coupled with safe and efficacious medical device performance led to FDA clearances and the introduction of novel products.

## Conclusion

The escalation of incidence and awareness of mTBI/concussion due to the Global War on Terror and the strong American predilection towards competitive sports created an urgent need for innovation in an area within medical technology specifically fraught with failure. Through long-term PPP between government, academia, and industry, which focused on clinical precision and regulatory compliance, a novel medical product category was created, and a unique FDA-cleared medical product addressing this substantial societal need was developed and introduced to the market. The partnership leveraged both public and private needs to achieve the goal of an FDA clearance of an mTBI device utilizing a novel technology. The utilization of the product in civilian life as a diagnostic adjunct in such venues as urgent care centers, university sports teams, concussion clinics, and hospital EDs demonstrates the results of this non-preferential PPP. Several important lessons emanated from this situation, including: defining the primary public health objective among all parties as the driving force for new product introduction; the importance of the government–academia–industry PPP triad with a “collaboration towards solutions” QbD mindset to assure evidence-based clinical validity with regulatory compliance; the development of device comparators and integration of measurements into a statistical and FDA pathway; and the utility of top-down, flexible, practical action while operating within governmental guidelines and focusing on patient safety.
